# Cerebral Venous Thrombosis in the Setting of Malignancy: Case Report and Review of the Literature

**DOI:** 10.1155/2020/8849252

**Published:** 2020-09-17

**Authors:** Constantine N. Logothetis, Charles Pizanis

**Affiliations:** ^1^Department of Internal Medicine, University of South Florida Morsani College of Medicine, 17 Davis Blvd, Suite 308, Tampa, FL 33606, USA; ^2^Division of Hospital Medicine Department of Internal Medicine, University of New Mexico School of Medicine, University of New Mexico, MSC10 5550, Albuquerque, NM 87131, USA

## Abstract

Cerebral venous thrombosis (CVT) is a rare condition that can be difficult to diagnose due to its vague and nonspecific symptoms. It is even more unusual to identify CVT in association with malignancy. Given the rarity of this disease, treatment and management of CVT in the setting of malignancy is not well defined. This case report and review of the literature addresses the epidemiology, pathophysiology, and medical treatment for malignancy-related CVT.

## 1. Introduction

Venous thromboembolism (VTE) is a common complication of cancer and cancer-related treatment. Among the possible VTEs that can occur are thromboses of the dural sinuses or cerebral veins. Cerebral venous thrombosis (CVT) is an uncommon clinical entity and is often difficult to identify due to its vague and nonspecific findings. Management of CVT can similarly pose a challenge for clinicians given the risk of bleeding complications with treatment. This case highlights a patient whose diagnosis of CVT was initially missed as was the underlying etiology of the disorder. This article reviews the epidemiology, pathophysiology, and medical management of CVT in patients with malignancy.

## 2. Case Presentation

**Figure 1:**
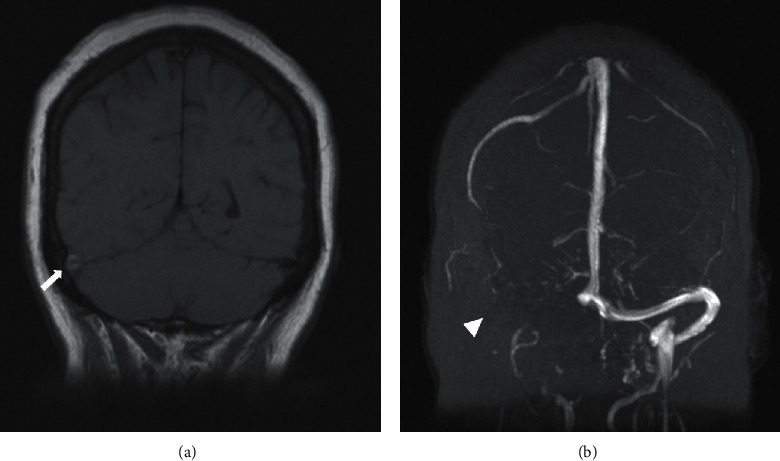
Magnetic resonance imaging demonstrating flow void at right transverse sinus (arrow) and absence of contrast (arrowhead) along transverse sinus consistent with transverse sinus thrombus.

A 60-year-old woman with a medical history of type 2 diabetes mellitus, papillary thyroid cancer status post resection, and hypertension presented to a referring hospital with worsening headaches and visual disturbances. For four months prior to her presentation, she had experienced intermittent unilateral, pulsating headaches as well as visual disturbances in the right eye. Upon arrival to the emergency department, she was febrile and noted to have meningismus. CBC revealed white blood cells at 9,800/L, hemoglobin at 12.9 g/dL, and platelets at 223,000/uL. Lumbar puncture was performed with a cerebrospinal fluid (CSF) profile demonstrating colorless fluid, total nucleated cells 43/mm^3^, red blood cells 4/mm^3^, glucose 134 mg/dL, total protein 50 mg/dL, and an opening pressure of 21 cm H20. CSF herpes simplex virus 1/2, West Nile virus, cytomegalovirus, enterovirus, varicella zoster virus PCRs, venereal disease research laboratory (VDRL), and coccidioides antibodies were negative or nonreactive, respectively. Serum HIV 1/2 antibodies, QuantiFERON-TB Gold, and autoimmune panel consisting of antinuclear antibody, rheumatoid factor, anti-mitochondrial antibody, and macrophage 2 antibodies were also nonrevealing. Magnetic resonance imaging (MRI) of the brain was obtained at the time which showed acute-to-subacute thrombosis of the right transverse sinuses (Figure 1). Given her constellation of symptoms and negative infectious and autoimmune workup, she was initially treated for aseptic meningitis and discharged home with warfarin anticoagulation for the thrombosis. Her symptoms did not fully resolve, however, and she was readmitted one week later to the hospital for further evaluation and workup.

On readmission, her CBC demonstrated elevated white blood cells at 17,000/L (38% polymorphonuclear cells, 34% lymphocytes, and 23% variant lymphocytes). Due to the presence of variant lymphocytes noted at that time, a peripheral blood smear was obtained which revealed precursor B-cells and 30% circulating blasts consistent with B-cell acute lymphoblastic leukemia (ALL). A repeat lumbar puncture was performed with CSF profile demonstrating total nucleated cells 728/mm^3^, red blood cells 0/mm^3^, glucose 93 mg/dL, and total protein 42 mg/dL. CSF flow cytometry demonstrated 97% blasts expressing CD34, CD19, bright CD10, and dim CD33 consistent with CNS involvement. After consultation with hematology, the patient was started on the ALL202 protocol with cyclophosphamide, daunorubicin, vincristine, methotrexate, cytarabine, and imatinib (given Ph chromosome positivity) as well as intrathecal methotrexate. During her hospitalization, her dural sinus thrombosis was treated with therapeutic-dosing enoxaparin at 1.5 mg/kg/day (100 mg).

During the course of her ALL treatment, the patient experienced repeated episodes of thrombocytopenia with clinical concerns for subarachnoid hemorrhage. After a year and a half of anticoagulation with enoxaparin, a follow-up MRI was obtained which demonstrated stability in the thrombosis since initial diagnosis. Because of the chronicity of the thrombus and the ongoing bleeding concerns, enoxaparin was discontinued. The patient underwent subsequent brain imaging during the course of her ALL treatment which did not demonstrate any increase in size of the dural sinus thrombosis despite lack of anticoagulation.

## 3. Discussion

Due to the rare nature the disease, there is little literature on the epidemiology, pathophysiology, and management of malignancy-related CVT. The prevalence of CVT in patients with malignancy is believed to be approximately 0.3% [1, 2]. Malignancy-related thromboses of the dural sinus or cerebral veins are thought to represent roughly 7.4% of all CVT cases, with cancer increasing risk for CVT roughly 5-fold[3]. Hematologic malignancies appear to be implicated more so than solid organ malignancies; however, the underlying reasons for this are not completely understood [4]. CVT has also been described in the setting of antineoplastic therapy. Several agents have been reported in the development of this condition including bevacizumab and lenalidomide [1, 5].

### 3.1. Pathophysiology

The pathophysiology behind the development of CVT is complex and follows Virchow's triad. While more clearly described in the setting of infection wherein localized inflammatory processes related to septic emboli, thrombi formation, and localized infection are thought to drive thrombus formation, the pathogenesis of CVT in the setting of malignancy is not well defined [6]. Several potential mechanisms have been proposed including direct tumor compression, tumor invasion, and the hypercoagulable state of malignancy [2, 7, 8]. In the absence of tumor compression or invasion, malignancy-related prothrombotic states are thought to drive development of VTE via a variety of mechanisms including a tumor's ability to increase the expression of tissue factor (TF), cancer procoagulant, and inflammatory cytokines as well as to downregulate the expression of thrombomodulin and the protein C system [9].

### 3.2. Medical Management

The treatment strategy for patients with malignancy-related CVT can be challenging. Similar to other causes of CVT, malignancy-related CVT carries risks of hemorrhage and hematoma formation. Moreover, patients with malignancy frequently experience thrombocytopenia and other coagulopathies as a result of the cancer and/or cancer therapies, making treatment decisions even more difficult. Lack of treatment, however, can lead to thrombus propagation, neurologic sequelae, and death [4]. As such, an individualized approach to treatment is recommended.

Much like other VTEs, the medical management of CVT primarily consists of systemic anticoagulation to reduce the risk of thrombosis growth and to facilitate vessel recanalization. Several agents have been described in the treatment of CVT including heparin, low-molecular weight heparins (LMWHs), vitamin-K antagonists (VKAs), and direct oral anticoagulants (DOACs). In 2011, the American Heart Association (AHA) and American Stroke Association (ASA) released a set of guidelines on the diagnosis and management of CVT. Contained within the recommendations were guidelines on acute as well as long-term treatments. Acute treatment options included heparins, LMWHs, and VKAs with VKAs being the primary recommendation for long-term treatment [4]. Additionally, the European Academy of Neurology (EAN) released similar recommendations in 2017 [10]. Notable differences include the recommendation of LMWHs over heparin in the acute treatment for CVT based in part on a more recent randomized controlled trial suggesting better recovery with LMWH [11]. Specific guidelines were limited, however, for malignancy-related CVT in both societal recommendations.

The existing literature on agent of choice for cancer-related CVT is primarily found in case reports. Several reports have been published demonstrating safety and positive outcomes of various anticoagulation regimens including heparin, LMWHs, and VKAs (Table 1) [1, 5, 12, 13–17, 18–31]. LMWHs have for several years been recommended over VKAs in malignancy-related VTE leading to the question of their superiority in malignancy-related CVT [32]. To date, however, no prospective trials evaluating LMWHs over VKAs have occurred. Despite these established recommendations for malignancy-related VTE elsewhere, specific recommendations for treatment of malignancy-related CVT were neither elaborated upon in the 2011 AHA/ASA nor the 2017 EAN CVT treatment guidelines [4, 10].

The emergence of the direct oral anticoagulants (DOACs) in the treatment of malignancy-related VTE is a new focus of great interest with multiple studies and societal recommendations supporting DOAC use to treat DVT and pulmonary embolism [33–36]. The role of DOACs in the treatment of CVT, however, is unclear [37]. In one randomized controlled trial examining the safety and efficacy of dabigatran versus dose-adjusted warfarin in patients with CVT, dabigatran was at least as safe, if not safer, than warfarin with regard to major bleeding risk. Neither group experienced recurrent VTE, and both had similar degrees of recanalization (60.0% in the dabigatran group and 67.3% in the warfarin group). Neither noninferiority nor superiority of either therapy could be demonstrated because of limited sample size [38].

Two case series exist describing the use of DOACs beyond the acute phase of treatment for CVT, one of which contained one patient with malignancy and CVT [39, 40]. In these series, DOACs appeared to be associated with favorable outcomes without significant bleeding concerns though absence of recanalization in the patient with malignancy was noted. Neither the Hokusai VTE Cancer Investigators nor the SELECT-D trials investigating DOAC treatment of malignancy-related VTE included patients with CVT. Given also the association of venous hemorrhage with CVT, use of DOACs may pose a clinical issue given limited (albeit growing) options for reversal agents [4]. In the 2017 European Academy of Neurology guidelines, DOAC therapy was not recommended for CVT based on low quality of evidence with a high risk for bias [10]. Further research is, therefore, needed into the efficacy and safety of DOACs for the management of malignancy-related CVT.

### 3.3. Duration of Therapy

The main goal of treatment beyond the acute therapy for CVT is prevention of thrombosis recurrence. New thrombosis following CVT has been reported as high 6.5% annually [41]. Optimal duration of long-term therapy to prevent recurrent CVT is often a question [42]. In the 2011 AHA/ASA and 2017 EAN CVT guidelines, a 3–12 month or indefinite anticoagulation duration was recommended depending on reversibility of the underlying etiology [4, 10]. Provoked CVT with a transient risk factor may be treated for 3–6 months, while unprovoked CVT may be treated for 6–12 months. Other authors have suggested ranges of weeks to several months or durations based on radiologic resolution of the thrombus. In the setting of malignancy, however, in which the provoking event may not be reversed prior to suggested end of therapy, an individualized decision should be made weighing risks of ongoing treatment as well as the risk for recurrence.

Currently, there are no consensus guidelines for the specific management of malignancy-related CVT with a majority of evidence for treatment found in case reports or extrapolated from guidelines focused on nonmalignant CVT. At this time, there is not a clearly superior anticoagulant for the management of malignancy-related CVT though it may be reasonable to consider LMWHs over VKAs given existing data on LMWH superiority in preventing VTE recurrence in patients with malignancy. DOAC therapy for malignancy-related CVT is not recommended at this time given lack of studies of efficacy and safety though they may emerge as future treatment options. Optimal treatment duration is similarly unclear though should be extended beyond acute treatment for CVT barring contraindications.

## 4. Conclusion

As described in this case, anticoagulation was administered upon the initial diagnosis of CVT. Given ongoing malignancy, the patient was treated with therapeutic-dosing enoxaparin for nearly two years which was eventually terminated after the complications of anticoagulation were thought to outweigh the risk of thrombus recurrence. While malignancy-related CVT is a rare phenomenon, further prospective study is warranted to establish firmer guidelines for anticoagulation. Until this occurs, treatment and duration should remain individualized. Data to date support the use of heparin, LMWH, and warfarin in the treatment of malignancy-related CVT. DOAC efficacy and safety are unclear at this time for the treatment of malignancy-related CVT though should be a focus of future investigation.

## Figures and Tables

**Table 1:** Review of the literature describing sinus thromboses in malignancies.

Reference	Number of patients	Cancer	Anticoagulant	Duration of therapy	Outcome
Oda et al.[15]	1	Stage IV lung adenocarcinoma	Heparin, warfarin	4 weeks	Radiographic resolution at 4 weeks
Iqbal and Sharma[13]	1	Metastatic colorectal adenocarcinoma	LMWH, warfarin	5 months	Clinical improvement in symptoms at 5 months
Mutreja et al.[14]	1	JAK2V617F mutation-positive hematolymphoid malignancy	Heparin, warfarin, dabigatran	21 days	Death at 21 days
Tuncel et al. [16]	1	Advanced-stage small cell lung cancer	LMWH	N/A	Clinical improvement at 1 month
Marvin et al. [3]	1	Metastatic renal cell carcinoma (brain metastases)	Heparin	N/A	Radiographic resolution and death at 4 months (unknown cause)
Shimizu et al. [17]	1	Tongue cancer	Heparin	N/A	Clinical and radiographic improvement
Akai et al. [18]	1	Malignant melanoma with meningeal dissemination	Urokinase, heparin	24 hours	Recanalization of straight sinus
Ho et al. [19]	1	Acute lymphoblastic leukemia	Heparin, warfarin	N/A	Radiographic resolution at 20 days
Vargo et al. [1]	1	Anaplastic gemistocytic astrocytoma	Heparin, enoxaparin	N/A	Stabilization and interval improvement on MRI
Eudo et al. [5]	1	Multiple myeloma	LMWH	N/A	Clinical improvement in symptoms at 1 month
Li et al. [20]	1	Grade 2, stage IIIC serous ovarian cancer	Enoxaparin	5 weeks	Death at 5 weeks
Chang et al. [21]	1	Acute myeloid leukemia, M1	Enoxaparin, warfarin	N/A	Clinical improvement in vision
Wang et al. [22]	1	Acute lymphoblastic leukemia	Enoxaparin	7 months	Clinical and radiographic improvement at six months
Iurlaro et al. [23]	9	Breast cancer x2, gastric cancer, NHL x3, HL, kidney cancer and melanoma, and rhinopharyngeal cancer	Unfractionated , LMWH	N/A	Based off of modified Rankin scale (mRS): complete recovery in 4 patients (mRS 0), mild sequelae in 4 patients (mRS 1-2), and moderate sequelae in one patient (mRS 3)
Karam and Koussa [24]	2	Germ-cell carcinoma of the testis; poorly differentiated pericardial carcinoma	Unknown anticoagulation; heparin	20 days	Clinical and radiographic improvement at 1 month; unknown outcome
Ross et al. [25]	4	T-cell ALL; pre-B-cell ALL; ALL; pre-B-cell ALL	LMWH; unfractionated heparin, LMWH, warfarin; LMWH; LMWH	7 months; n/a; 3 months; 4 months	Radiographic partial recanalization; clinical improvement in symptoms; complete resolution on imaging at 1 month; resolution on imaging at 1 month
Song et al. [26]	1	Acute promyelocytic leukemia	LMWH, warfarin	N/A	Radiographic recanalization of straight sinus with continued occlusion of transverse and confluens sinuum at 1 month
Imamura et al. [27]	2	B-precursor ALL; T-cell type NHL	No anticoagulation; LMWH; ATIII concentrate supplementation	N/A; N/A	Death at 40 days; complete neurologic recovery
Lee et al. [28]	1	Acute lymphoblastic leukemia	Heparin, warfarin	N/A	Radiographic recanalization of the superior sagittal sinus
Ciccone et al. [29]	1	Acute promyelocytic leukemia	Unfractionated heparin, warfarin		Complete recovery from the coma and almost complete resolution of the sinuses thrombosis.
Motohashi et al. [30]	1	Acute myeloid leukemia	Heparin, warfarin	N/A	Recanalization of straight sinus but continued occlusion of the right transverse and sigmoid sinuses. No neurologic sequelae at 10 months
Corso et al. [31]	2	Ph + ALL; Ph + ALL	Heparin; heparin	N/A; N/A	Radiographic resolution of thrombus; progressive but slow resolution of symptoms
